# Altered modulation of gamma oscillation frequency by speed of visual motion in children with autism spectrum disorders

**DOI:** 10.1186/s11689-015-9121-x

**Published:** 2015-08-10

**Authors:** Tatiana A. Stroganova, Anna V. Butorina, Olga V. Sysoeva, Andrey O. Prokofyev, Anastasia Yu. Nikolaeva, Marina M. Tsetlin, Elena V. Orekhova

**Affiliations:** Autism Research Lab, MEG-Center, Moscow State University of Psychology and Education, Sretenka 29, Moscow, 107045 Russian Federation

**Keywords:** ASD, Visual gamma oscillation frequency, Stimulus velocity, Oblique line orientation threshold

## Abstract

**Background:**

Recent studies link autism spectrum disorders (ASD) with an altered balance between excitation and inhibition (E/I balance) in cortical networks. The brain oscillations in high gamma-band (50–120 Hz) are sensitive to the E/I balance and may appear useful biomarkers of certain ASD subtypes. The frequency of gamma oscillations is mediated by level of excitation of the fast-spiking inhibitory basket cells recruited by increasing strength of excitatory input. Therefore, the experimental manipulations affecting gamma frequency may throw light on inhibitory networks dysfunction in ASD.

**Methods:**

Here, we used magnetoencephalography (MEG) to investigate modulation of visual gamma oscillation frequency by speed of drifting annular gratings (1.2, 3.6, 6.0 °/s) in 21 boys with ASD and 26 typically developing boys aged 7–15 years. Multitaper method was used for analysis of spectra of gamma power change upon stimulus presentation and permutation test was applied for statistical comparisons. We also assessed in our participants visual orientation discrimination thresholds, which are thought to depend on excitability of inhibitory networks in the visual cortex.

**Results:**

Although frequency of the oscillatory gamma response increased with increasing velocity of visual motion in both groups of participants, the velocity effect was reduced in a substantial proportion of children with ASD. The range of velocity-related gamma frequency modulation correlated inversely with the ability to discriminate oblique line orientation in the ASD group, while no such correlation has been observed in the group of typically developing participants.

**Conclusions:**

Our findings suggest that abnormal velocity-related gamma frequency modulation in ASD may constitute a potential biomarker for reduced excitability of fast-spiking inhibitory neurons in a subset of children with ASD.

**Electronic supplementary material:**

The online version of this article (doi:10.1186/s11689-015-9121-x) contains supplementary material, which is available to authorized users.

## Background

Autism spectrum disorders (ASD) are a group of neurodevelopmental disorders characterized by impaired social interaction and communication, repetitive behaviors, and restricted interests. ASD is now viewed as a heterogeneous set of pathological conditions, which can be caused by various genetic, epigenetic, and environmental factors [1]. Given a large diversity of molecular, cellular, and network mechanisms converging on the same ASD behavioral phenotype, many researchers currently agree that further progress in ASD research depends on the efforts undertaken to reduce heterogeneity by subsetting individuals with ASD according to traits that may be related to underlying pathophysiology [2]. In addition, neural abnormalities associated with ASD might be more adequate targets for diagnostic and therapeutic advancement than behavioral phenotypes [3].

Emerging evidence suggests that imbalance between excitatory glutamate and inhibitory gamma-amino-butyric-acid (GABA) neurotransmission may form one of the several common pathways for multiple molecular-genetic abnormalities in ASD [4, 5]. In particular, animal research as well as the studies of postmortem brain tissue of people with ASD highlighted the role of GABAergic inhibitory interneurons for appearance of ASD phenotype [6, 7].

A plausible neurophysiological model explaining changes in brain functioning caused by abnormal activity of specific type of GABAergic interneurons—fast-spiking (FS) parvalbumin-expressing (PV^+^) cells—is an aberration in gamma oscillations (30–120 Hz) in cortical networks [8]. Gamma oscillations act to temporally coordinate firing of excitatory cortical pyramidal neurons both within local and distant neural networks, and such coordination is thought to be the key component of information processing in distributed cortical assemblies [9]. In humans, gamma oscillations induced by certain functional loads have been implicated in a wide range of brain functions, such as attention [10], visual perception [11], and motor control [12, 13]. Pathological modifications of oscillatory gamma response properties may therefore account for cognitive, perceptual, and motor dysfunctions that have been frequently observed in individuals with ASD since infancy [14].

How the disturbances of cortical gamma activity in neurodevelopmental disorder can give rise to deviations from typical developmental trajectory of a particular brain function is not yet clear. It would be therefore important to investigate whether impaired modulation of gamma oscillations by specific functional load signifies deficient ability of cortical networks to be effectively mobilized by sensory input or attentional demands.

Vision is one of the particularly interesting functional domains in this respect. Indeed, center–surround antagonism mediated by inhibition in the visual cortex is critically important for basic visual functions including orientation discrimination [15], contour detection [16], figure–ground segmentation, and perception of motion [17, 18]. On the other hand, there is a growing body of evidence showing that atypical basic perceptual skills are at least a concomitant and possibly the cause of some core behavioral signs and symptoms in a subset of ASD individuals (for review, see [14]). However, only a few studies investigated EEG gamma oscillations triggered by perception of visual stimuli in children with autism [19–22], and none of them studied the relationship between gamma response and psychophysical measures of basic visual functions.

Until now, the key obstacle toward the use of this potential biomarker of inhibitory dysfunction in cortical networks and relating it to behavior and visual functions in ASD the lack of the experimental paradigms that consistently provoked measurable visual gamma response in EEG and/or magnetoencephalography (MEG) recordings. Non-invasive detection of these low amplitude brain oscillations is complicated due to the presence of strong muscular and ocular artifacts, which overlap the same frequency range as the gamma oscillation [23, 24]. Therefore, artifact-free measurement of gamma-band oscillations requires a combination of highly optimized experimental paradigms coupled with reliable methodology of gamma response detection.

The recently developed “moving annular grating” paradigm [25] was proved to very effectively induce visual gamma oscillations in EEG and MEG recordings. Several studies have demonstrated that in adults, contracting and/or expanding annular gratings induced reliable MEG-recorded high-frequency (60–90 Hz) gamma oscillations (HGO) in the visual cortex [25–27]. Moreover, results of Edden and colleagues [28] suggest a causal link between HGO response frequency and GABAergic pathways (but see [29]). Animal studies suggest that abnormalities of both inhibitory GABAergic transmission and excitatory NMDA-mediated transmission on the inhibitory FS PV^+^ interneurons may affect gamma frequency [30, 31]. Hence, studies of visually induced high-frequency oscillations in individuals with ASD may appear useful to reveal the subtype of the disorder most strongly associated with abnormalities in inhibitory network of the visual cortex. This paradigm, however, has not been yet applied to investigate adults or children with ASD.

We hypothesized that the altered excitation/inhibition balance (E/I balance) within the visual cortical network in a subset of ASD subjects could affect both the induced gamma oscillation in the visual cortex and subjects’ ability to perceive visual information.

To check this hypothesis, we applied the moving annular grating paradigm and used MEG—the non-invasive methodology that is more sensitive than EEG to the cortical electromagnetic sources oscillating at frequencies in the higher gamma-band range [32].

To ensure functionality of the HGO response to moving annular gratings, we introduced an important modification into the paradigm by changing stimulus velocity from 1.2 up to 6.0 °/s. The animal studies demonstrated a systematic increase of HGO frequency in the primary visual cortex with increasing stimulus velocity [33, 34]. Our recent study has shown that changing speed of visual motion effectively modulates gamma frequency also in children [35].

In this study we, for the first time, attempted to characterize frequency modulation of induced gamma oscillatory response in children with ASD. If found, the gamma frequency abnormality could appear a reliable biomarker of altered E/I balance in cortical networks. Firstly, the previous studies employing the moving annular grating paradigm showed that frequency of induced gamma response is an individually stable and genetically determined trait [27, 36], thus ensuring usefulness of its possible application for clinical testing. Secondly, in EEG and MEG recordings, gamma frequency modulation could be a more direct and accurate functional measure than the gamma power, as the latter is affected by individual variations in non-physiological variables, such as convolution of cortical tissue generating electromagnetic signal [37] or degree of EEG/MEG signal contamination by muscle activity [23].

To investigate perceptual consequences of putative visual gamma response abnormalities in ASD, we have tested in our subjects the line orientation discrimination thresholds in a complementary psychophysical session. The ability to discriminate line orientation depends on GABA-mediated lateral inhibition in the primary visual cortex [38]. It has been recently shown that in healthy adults, the orientation discrimination thresholds for obliquely oriented lines correlated negatively with concentration of inhibitory mediator GABA in the visual cortex [28]. We for the first time examined the relationship between oblique line discrimination and visual gamma response in typical children and those with ASD.

## Methods

### Subjects

Twenty one boys with ASD aged 7–15 years (mean age 10.4 years; SD = 2.2) were recruited at rehabilitation centers affiliated with the Moscow University of Psychology and Education. All participants with ASD were assessed by the same experienced licensed psychiatrist. The diagnosis of ASD was based on the Diagnostic and Statistical Manual of Mental Disorder-V criteria as well as an interview with the parents/caregivers [39]. The inclusion criteria were the absence of known chromosomal syndrome (e.g., Down syndrome, fragile X syndrome) or comorbid neuropsychiatric disorder (e.g., epilepsy).

Twenty six age-matched (mean age 11.1 years; SD = 1.7) typically developing (TD) control boys were recruited from local schools by advertisements. The TD boys comprising the control group in this study were also included in our recent normative study of visual gamma oscillations [35].

To confirm validity of the diagnosis, parents of all the children were asked to fill in the Russian version of the autism spectrum quotient (AQ) for children [40] or adolescents [41]. The AQ results were available in 19 of 21 ASD and in 22 of 26 TD participants. The majority of parents were also asked to fill in the “lifetime” version of the Social and Communication Questionnaire (SCQ; [42]). The sensitivity and specificity of the SCQ-lifetime and AQ questionnaires in our sample are reported in Additional file 1: Table S1. The results from both questionnaires were in good agreement with the clinical diagnosis.

The intellectual abilities of children have been assessed with the Kaufman Assessment Battery for Children К-АВС II [39]. The IQ scores were available in 19 of 21 ASD and 25 of 26 TD participants. Information on participant’s age, IQ, and AQ scores and the number of the participants tested are summarized in Table 1. All children had normal or corrected to normal vision according to available medical records.

**Table 1:** Demographic information: mean ± sd (range)

	ASD (*N* = 21)	TD (*N* = 26)
AGE (years)	10.4 ± 2.2 (7.7–15.3)	11.1 ± 1.7 (6.9–14.1)
Sequential IQ^a^	94.3 ± 15.4 (63–127)	100.2 ± 1.2 (88–121)
Simultaneous IQ^a^	95.9 ± 16.2 (71–144)	120.7 ± 13.2 (91–150)*
Mental processing composite^a^	93.9 ± 18.3 (59–127)	117.6 ± 12.3 (92–141)*
Child AQ^b^	87.9 ± 10.6 (73–119)	56.3 ± 15.07 (32–85)*

Asterisks denote significant difference between ASD and TD group, *p < 0.05

^a^IQ was available in 19 of 21 ASD and in 25 of 26 TD participants

^b^AQ was available in 19 of 21 ASD and in 22 of 26 TD participants

The previous studies suggest significant sex differences in etiological factors and the biological time course of ASD [43]. The gender ratio in ASD is highly biased toward boys [44]. Since the small number of participants who could be recruited for this study did not allow us to test for the effect of gender, we included only male participants. This limits the generalizability of our results.

The study was approved by the local ethics committee of the Moscow University of Psychology and Education and was conducted following the ethical principles regarding human experimentation (Helsinki Declaration). A written informed consent was obtained from a parent/guardian of each child.

### Experimental procedure

During the MEG recording, subjects sat in a magnetically shielded room (“AK3b”, Vacuumschmelze GmbH, Hanau, Germany) with the head resting securely against the helmet-shaped surface of the helium Dewar.

The stimulus used in this study was a black and white contracting annular grating having spatial frequency of 1.66 cycles/° and a diameter of 18 ° of visual angle. The stimuli were shown to the participants using Presentation software (Neurobehavioral Systems Inc., USA) via a computer with 60 Hz refresh rate and were back-projected on a translucent white projection screen located 1.1 m in front of the participants. The stimuli were presented in the center of the screen at three contraction velocities (1.2, 3.6, 6 °/s), referred below as low, medium, and fast. In order to minimize visual and mental fatigue, the test stimuli were interspersed with 67 short (3–6 s) animated movies. A small white fixation cross on the black background was presented in the center of the screen between the stimuli.

Each trial began with presentation of a fixation cross. The participants were instructed to constantly maintain their gaze on the cross when it was present. In 1200 ms, the annular grating contracting with one of the three velocities was presented for a period that varied randomly between 1200 and 3000 ms and was followed by a static stimulus picture. The participants were instructed to press a button as quickly as possible after termination of the stimulus motion. The subjects’ response was followed by appearance of the fixation cross and presentation of the new stimulus. The response hand was counterbalanced across experimental blocks. If the response latency exceeded individually calculated maximum (median response time + 3SD determined in the training session), the stimulus was substituted by discouraging message “too late!” remaining on the screen for 2000 ms, and then the new trial began.

The training session preceded the main experiment and served to familiarize participants with the task and to measure the median response time in order to adjust the maximal response interval in the main experiment. The training session was fully identical to the experimental one, but contained only 20 trials and imposed no time limit on response.

The experimental session included three blocks of trials. Each stimulus velocity was presented 30 times within each block in a random order resulting in 90 trials per stimulus velocity.

Response time (RT) as well as commission and omission errors were measured for all but one TD subject (due to technical failure) and all but two ASD subjects, who did not follow the instruction to press a button, but still were looking at the screen.

### MEG recording

Neuromagnetic activities were recorded with the helmet-shaped 306-channel detector array (“Vectorview”, Neuromag Elekta Oy, Helsinki, Finland), which comprised 102 identical triple sensor elements. Each sensor element consisted of two orthogonal planar gradiometers and one magnetometer coupled to a multi-SQUID (superconducting quantum interference device). In this study, the data from 204 planar gradiometers were used for analyses because they provide an optimal signal-to-noise ratio for spatially restricted current sources such as occipital gamma generators.

Prior to the MEG session, the positions of HPI coils were digitized together with fiducial points using the 3D digitizer “FASTRAK” (Polhemus, Colchester, VT) and were further used to assess a subject’s head position inside the MEG helmet every 4 ms. The spatiotemporal signal space separation method (tSSS) method [45] implemented by “MaxFilter” (Elekta Neuromag Oy software) was used to suppress interference signals generated outside the brain. Head movement compensation was used to convert the data to the standard head position (x = 0 mm; y = 0 mm; z = 45 mm) across all time points and experimental blocks.

Four electrodes were placed at the outer canti of the eyes and above and below the left eye and used to record electrooculogram (EOG). The high-pass filter of 0.1 Hz was used for the EOG recording. The electrocardiogram (ECG) was recorded using standard V6–V2 leads. The MEG signals were recorded with a band-pass filter of 0.03–330 Hz, digitized at 1000 Hz, and stored for offline analysis.

### MEG data preprocessing

The correction of vertical eye movements and ECG artifacts was performed for continuous data in Brainstorm (http://neuroimage.usc.edu/brainstorm [46], using the SSP algorithm [47, 48].

The subsequent analyses were done using the SPM12 toolbox (http://www.fil.ion.ucl.ac.uk/spm [49]). The pipeline was optimized for analysis of velocity-driven HGO time–frequency modulations through defining velocity-specific spectral power changes over the occipital cortex.

The averaging epoch started 500 ms prior to stimulus onset and lasted until 1200 ms post-stimulus. The MEG epochs containing strong muscle artifacts were excluded by thresholding the mean absolute values of high-frequency signal. The threshold was set at 5 standard deviations from the absolute amplitude of the 70 Hz high-passed signal averaged across channels. The remaining epochs were further visually inspected for the presence of artifacts, and the artifact-containing epochs were excluded manually. The average number of artifact-free epochs per condition was 79.6 (54–89) in the ASD group and 83.0 (56–90) in the control group and did not significantly differ between the groups or conditions (Table 2).

**Table 2:** Moving annular gratings paradigm: behavioral results and a number of epochs sampled for MEG analysis

	Slow velocity mean (SD)		Medium velocity mean (SD)		Fast velocity Mean (SD)	
	*ASD*	*TD*	*ASD*	*TD*	*ASD*	*TD*
Number of epochs	79.6 (7.3)	83.3 (5.5)	79.0 (7.5)	82.7 (7.3)	80.1 (7.1)	83.0 (6.6)
Reaction time (ms)^a^	509.9 (101.4)	436.4** (62.5)	474.9 (92.7)	414.6* (63.2)	463.9 (103.5)	407.3* (70.8)
Omission errors (% trials)^a^	2.2	2.5	2.1	1.8	1.2	2.2
Commission errors (% trials)^a^	0.36	0.55	0.44	0.61	0.17	0.87

Asterisks denote significant difference between ASD and TD group, *p < 0.05; **p < 0.01

^a^Behavioral results were available in 19 of 21 ASD and in 25 of 26 TD participants

For efficient spectral estimation, we used multitaper spectral analysis [50]. This method is based on pre-multiplying the data with a series of tapers optimized for producing uncorrelated estimates of the spectrum in a given frequency band. This sacrifices some of the frequency resolution, in a controlled manner, to increase signal-to-noise ratio. It does this by effectively multiplying the number of trials by the number of tapers used. We estimated the spectra in overlapping windows of 400 ms (shifted by 50 ms). The frequency resolution was set to the inverse of the time window (2.5 Hz) for up to 25 Hz, then 0.1 times the frequency for 25–50 Hz, and then to a constant 5-Hz resolution. These settings resulted in a single taper being used for 2.5–30 Hz, two tapers for 32.5–42.5 Hz, and three tapers for 45 Hz and above. The resulting time–frequency images had no discontinuities, thanks to the continuous frequency resolution function.

The epoched time–frequency data were averaged using a robust averaging procedure [51, 52]. To reduce inter-subject variability and to normalize power changes across different frequency bands, the –500 to 1200 ms power was log transformed and baseline corrected using the period from –500 to –100 ms before stimulus onset as the baseline (LogR option in SPM). Planar channels were then combined by adding time–frequency data for pairs of channels corresponding to orthogonal sensors at the same location. Based on the previous neuroimaging studies of visual HGO response to moving annular gratings in human subjects [25, 26], we expected the response to be restricted by MEG sensors overlaying the occipital lobe. Therefore, we used the posterior planar gradiometers for subsequent analysis.

### Group analysis of MEG data

Our prior study in the TD children [35] has shown that the faster velocities of visual motion induce gamma oscillatory response at higher frequencies in the same way as it has been shown in the animal studies [34]. We hypothesized that these velocity-related frequency modulations may be compromised in children with ASD. For identification of velocity-specific power changes at each of the three stimulus velocities, we performed statistical analysis of the stimulus-related spectral changes to detect significant differences between velocities. The group-level analysis, therefore, proceeded in the following steps.

First, we calculated average log-transformed baseline-normalized power (in dB) in 400 to 1000 ms post-stimulus window for each frequency bin within 50–120 Hz range. This time window displays strong sustained gamma response to visual motion and is not contaminated by stimulus onset response [25, 26]. In both groups of subjects and for all three velocities, the topographical maximum of the stimulus-related spectral power changes was found at planar gradiometers pair 2112/2113 overlaying midline of the occipital lobe.

Second, at the 2112/2113 gradiometer pair, we searched for velocity-specific frequencies where the baseline-normalized HGO spectral power for a given stimulus velocity exceeded that for the two other velocities, while properly corrected for the family-wise error using permutation approach. To correct for multiple comparisons, the baseline-normalized spectral power values at each frequency bin within 50–120 Hz range were randomly permuted 1000 times between the two datasets (e.g., low and medium velocity conditions) and the parametric one-sided *t* test was performed. Across all frequency bins, the maximal *t* value from each permuted dataset was taken when forming the empirical null distribution and comparing it with the original statistics [53]. The probability level was set at *p* < 0.05 for this comparison.

Velocity-specific frequencies were computed as an overlap between results of the two unidirectional pairwise comparisons. In the TD group, the distinct velocity-specific HGO frequencies were found for all three stimuli velocities. In the ASD group, the velocity-specific increase in gamma power was found only for the slow velocity.

For each stimulus velocity, we also compared HGO response strength between ASD and TD subjects across the whole spectrum of gamma power changes. The same permutation approach was used for the group comparison (Fig. 2, right panels).

### Assessment of individual velocity-specific peak frequency of HGO response

There is a substantial inter-individual variability in the frequency boundaries and sensor sites where MEG-recorded HGO response could be observed during moving annular grating perception even in healthy adults [26]. Depending on the subject or even on the electrode/sensor site, power augmentation at the higher-frequency part of the EEG/MEG spectrum (50–120 Hz) was reported across a wide range of frequencies. It is likely that the frequency and spatial variability of the visual HGO response in children is further inflated by age and—in the ASD participants—by variations in their pathological condition. The direct between-group statistical comparisons, therefore, may lead to false negative results due to individual variability of HGO response both in space and frequency. Considering that the main focus of our study was on the frequency modulations of HGO by velocity, we applied a more flexible approach to the definition of individual velocity-specific frequency of HGO response.

In response to each stimulus velocity, the majority of children in both groups demonstrated a prominent peak of spectral power changes within 50–120 Hz frequency range in at least one of the occipital sensors. In each subject and for each stimulus velocity, we calculated individual velocity-specific peak frequency (VSPF). Considering possible individual variations in head position and cortical morphology, the individual VSPF was always defined at the gradiometer pair where the maximal HGO response in 50–120 Hz range was observed in a particular subject for a particular velocity. To ensure that the individual values comply with the group HGO response, the search for the individual gamma VSPF was limited to a certain frequency window. This window was centered at the TD group velocity-specific gamma peak which overlapped or was closest to velocity-specific frequencies at the group level (62.5 Hz for the slow, 80 Hz for the medium, and 92.5 Hz for the fast velocity). The width of each velocity-specific window was set at the full width at half maximum (FWHM). This resulted in velocity-specific frequency windows of 52.5–77.5 Hz for the slow velocity, 55.0–87.5 Hz for the medium velocity, and 55.0–97.5 Hz for the high velocity. Inspection of the group response spectra (Fig. 2) shows that the resulting frequency windows were broad enough to include visual gamma responses in both the TD and ASD groups. The individual spectra were smoothed by a three points moving average filter. After the smoothing, the algorithm searched for the peaks of the baseline-normalized power within the velocity-specific windows. Given that a specified frequency window may cover several local maxima, the peak of the highest frequency was always taken from all the peaks that exceeded baseline value by two standard deviations. This approach makes explicit the assumption that there may be several spectral peaks in gamma response (see e.g., [34]) and is less susceptive to arbitrary detection of a local maximum. The VSPFs that complied with the above specified criteria for all three velocities were found in 16 ASD and 19 TD participants. Additional file 2: Table S2 summarizes demographic information on the subjects included in statistical analysis of VSPF.

### Statistical analysis of VSPF

To assess contribution of subjects’ age and IQ score into the inter-individual variation of gamma VSPF at each stimulus velocity, we calculated Spearman non-parametric correlations separately in the TD and ASD groups. For between-group comparison of the VSPF values, we performed general linear modeling (GLM) analysis with the within-subject factor velocity (1.2, 3.6, 6.0 °/s) and between-subject factors group (TD and ASD) and age. We tested for the main effects as well as for interactions of velocity with the between-subject factors. The Greenhouse–Geisser correction for violation of sphericity assumption was applied. Significant interaction was followed up by planned comparisons.

### Psychophysical task

The oblique and vertical orientation discrimination thresholds were measured in two successive experimental sessions within 2 months after the MEG experiment. To measure the thresholds, we applied a two-alternative forced choice procedure similar to that used by Edden and colleagues [28]. The stimuli were presented on 19” W-LED “Nec Multisync EA192M-BK” monitor (resolution 1280 × 1024) controlled by a “Mobile Intel® 945GM Express” graphics chipset. Participants were asked to sit comfortably at 60 cm distance from the monitor. The distance from the monitor, vertical head position, and adequate task performance was controlled by an assistant who sat next to the subject. The room was completely dark, and a circular aperture (with diameter of 61 cm and inner empty circle of 13 cm) was placed over the monitor to remove all external orientation clues, such as those from the edges of the screen. Attention to the center of the screen has been facilitated by flashing a dot in the center of the screen, which appeared in the beginning of each trial. Each trial contained two circular gratings (diameter 7 °; spatial frequency 3 cycles/degree; contrast 100 %; mean luminance 3.3 lux) presented sequentially for 350 ms, with the time interval varying randomly from 400 to 600 ms. The orientation difference between the gratings was adjusted using two interleaved one-up two-down staircases that converged on 71 % correct performance. The orientation of the first grating was held fixed at 90 ° in the “vertical” condition and 45 ° in the “oblique” condition, and the second grating was rotated either clockwise or counterclockwise. The initial difference between the first and the second grating was 15 °. The initial step was 1 °, which was reduced to 0.4 ° after the second reversal and to 0.2 ° after the fourth reversals. Participants were asked to judge whether the second grating was rotated clockwise or counterclockwise compared to the first one. Responses were made using the keyboard. There were separate blocks, consisted of either vertical or oblique trials, with the block order counterbalanced across participants. The majority of the participants performed each block twice, and the final threshold was based on the average. Each block continued until both staircases completed seven reversals, typically lasting about 7 min. The threshold was computed by taking average over the reversals, excluding the first two of each staircase, and then over the two staircases.

To decrease the number of statistical comparisons, only the oblique orientation thresholds were analyzed in the present study as they were previously shown to correlate with frequency of visual gamma oscillations in neurotypical adults [28]. These oblique orientation threshold data were available for 23 TD participants and 14 participants with ASD. The number of children included into statistical analysis of VSPF was 16 and 13, respectively (see the Additional file 2: Table S2). The common logarithm of orientation discrimination threshold assessed in degrees was taken as a dependent variable in further analysis.

## Results

### Behavioral results

Behavioral data from the drifting annular grating task (Table 2) indicated no significant group or velocity effects or their interaction in terms of omission or commission errors (F(2,84) = 1.94; *p* = 0.15; F(2,84) = 1.60; *p* = 0.21). The reaction time was slower in the ASD than in TD group (F(1,42) = 7.2; *p* < 0.02), but no group × velocity interaction effect was detected (F(2,84) = 2.4; *p* = 0.10). Individuals with ASD are often referred to in the literature as being relatively slow and inaccurate in their perceptual–motor skills [54]—the finding that may reflect problems with attention focusing. Nevertheless, the lack of the interaction between group and velocity suggests that the paradigm equally engaged attention of TD and ASD subjects in each velocity condition, and that any interactions between group and velocity found for the neurophysiological variables cannot be explained by differences in attention.

### Velocity-specific MEG gamma responses

**Fig. 1:**
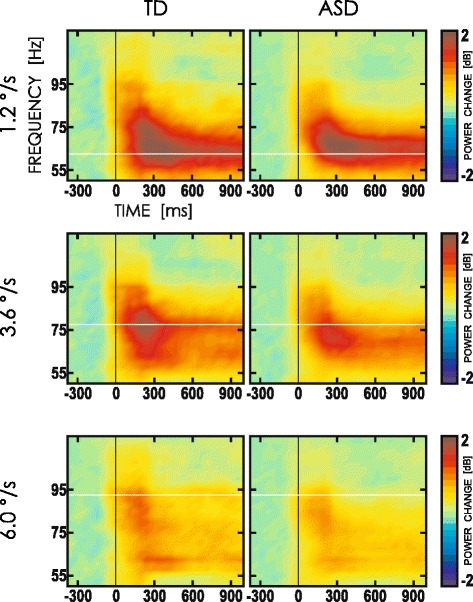
Visually induced gamma activity in the TD and ASD groups: grand-averaged time–frequency plots of HGO power changes elicited by gratings drifting at velocities of 1.2, 3.6, and 6.0 °/s. TF plots are shown for gradiometer’s pair MEG2112 and MEG2113 located at the scalp topographical maximum of gamma response. *Color scale* represents power changes in dB relative to pre-stimulus baseline. *Vertical line* marks the stimulus onset; *horizontal lines* correspond to the mean velocity-specific peak frequency (VSPF) in the TD group

**Fig. 2:**
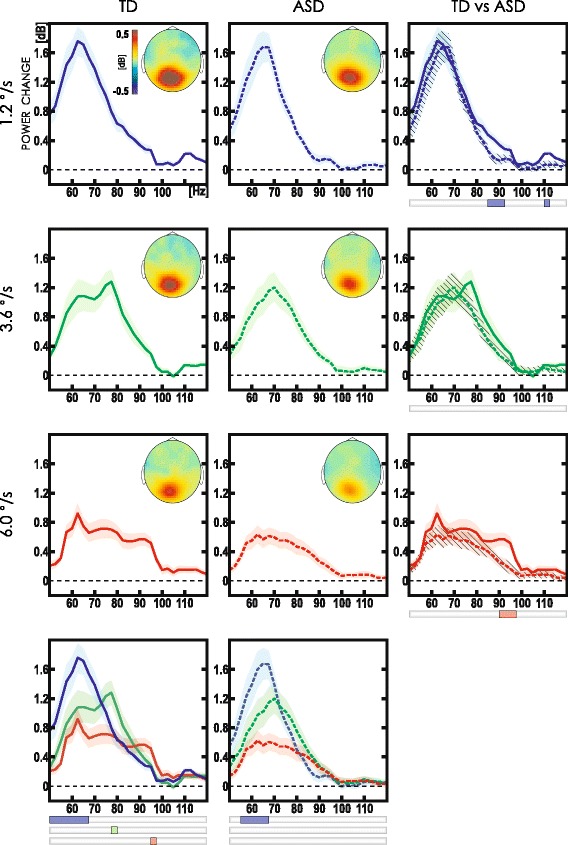
Grand average spectra of HGO response induced by drifting gratings in the TD and ASD groups. Spectra are shown for combined pair of MEG gradiometers that displayed maximal gamma response (MEG2112 and MEG2113). Horizontal axis—frequency (Hz), vertical axis—log-normalized power changes relative to baseline (dB). *Blue line*—1.2 °/s, *green line*—3.6 °/s, *red line*—6.0 °/s. *Solid* and *dashed lines* designate the TD and ASD group, respectively. *Shaded* and *striped shaded* areas represent standard error of the mean for the TD and ASD, respectively. Sensor level topographic maps of the high gamma-band (55–120 Hz, 0.4 to 1.2 s) activity in dB are shown in the upper left corner of the respective panel. Bottom panels show between-condition comparisons; *strips* below the plots indicate frequencies where power increase for the respective velocity exceeded that for the two other velocities (*p* < 0.05, FWE-corrected). Between-group comparisons for each velocity condition are shown in the rightmost panels. The *strips* designate significant group differences (*p* < 0.05, FWE-corrected). Note that the TD/ASD difference in velocity-specific HGO response is evident for the fast stimulus velocity (6.0 °/s) only. Although at the medium velocity (3.6 °/s) this difference was also significant at the uncorrected level as evident from the error bars, this effect did not survive FWE correction

Inspection of grand average time–frequency plots for power changes shows a robust increase of HGO power within 50–120 Hz frequency range at each stimulus velocity, in both the TD and ASD groups. This increase in high gamma power was sustained for the whole duration of the stimulus presentation (Fig. 1). The inserts in Fig. 2 show topographical distribution of the average HGO response power. As expected, the maximal HGO response clustered over the medial parieto-occipital cortex with a topographical maximum at the gradiometers pair 2112 + 2113 overlaying the occipital midline.

Importantly, the frequency characteristics of visual HGO response showed a clear divergence between the three stimulus velocities in the TD group where the velocity increase resulted in significant velocity-specific HGO response at higher frequencies (Fig. 2). In the ASD group, the peak frequency of the HGO response elicited by slow stimulus velocity almost precisely corresponded to that in the TD group, while for the medium and fast velocities, the velocity-specific HGO responses in the ASD group were not detected (Fig. 2, bottom panels).

### Group differences in gamma VSPF

**Fig. 3:**
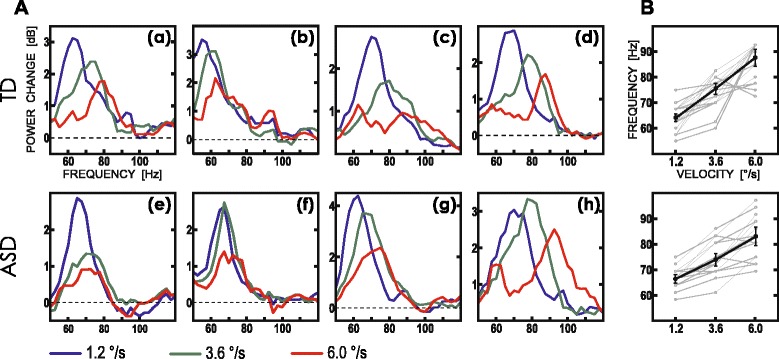
Examples of spectra of HGO power changes caused by drifting gratings moving at three velocities in individual subjects from the TD and ASD groups. **a** Individual spectra of HGO response. *Upper row*—TD subjects; *bottom row*—ASD subjects. See Fig. 2 for color designations. Although faster-moving gratings produce shift toward higher response frequency in individual subjects from both groups, the frequency shift is greater in TD individuals. Noticeable exception is the subject *(h)* from the ASD group. **b** Velocity-specific peak frequencies (VSPF) of the HGO response plotted as a function of stimulus velocity for individual participants (*thin lines*). The *thick lines* show group means for the TD and ASD subjects

The individual data revealed substantial variability in gamma VSPF, which varied between 57.5 and 92.3 Hz. The examples of individual visual HGO spectra in the TD and ASD participants are shown in Fig. 3. Previous studies reported inter-individual variations in gamma peak frequencies over a narrower range [25, 26, 36, 55]; however, these experiments used grating drifting at a fixed velocity close to the slowest velocity used in our experiment.

Overall, the individual VSPFs for all three velocities were detected in 19 out of 26 TD participants and in 16 out of 21 participants with ASD. In every individual subject, the gamma VSPF tended to be highest when he was presented with the gratings drifting at fastest velocity. In the TD group, the VSPF in response to the slow and fast velocities differed by 23 Hz on average, with a maximal difference of 37.5 Hz (Fig. 3). The mean standard error of the estimated oscillation frequency shift between low and fast velocity was 1.97 Hz. As evident from the individual HGO response spectra, the progressive shift in oscillation frequency was present also in participants with ASD, although the inter-individual differences in velocity-related frequency gain were greater in ASD than in TD subjects (e.g., compare subjects (e) and (h) in Fig. 3).

Furthermore, for each subject, we defined the “modulation range” of gamma VSPF as the difference of the VSPF values driven by the fast and slow velocities of the drifting grating.

**Fig. 4:**
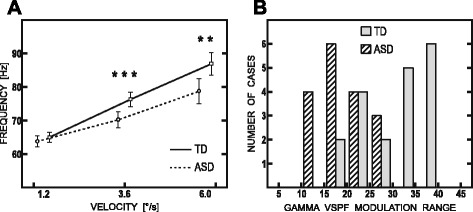
Velocity-related changes of HGO frequency in ASD and TD subjects. **a** GLM results for gamma VSPF. *Solid line*—TD; *dashed line*—ASD. *Asterisks* denote significant between-group differences: **p < 0.005; ***p < 0.001. **b** Frequency distribution of VSPF modulation range (i.e. difference in VSPF for the fast and slow stimulus velocities) in the TD (*white bars*) and ASD (*striped bars*) groups

The GLM analysis revealed a substantial between-group difference in velocity-related modulation of gamma VSPFs. The significant velocity by group interaction (F(2,64) = 4.40; ε = 0.88; *p* < 0.02) was mainly explained by significant between-group differences in gamma VSPF in case of the medium and fast velocities and absence of such differences in case of the slow velocity condition (Fig. 4a). The latter finding suggested that gamma VSPF modulation range was reduced in participants with ASD compared to control participants. Of note, the velocity by group interaction remained significant when we included IQ as a nuisance variable in the GLM analysis (F(2,60) = 4.3, ε = 0.88, *p* < 0.02) and further when we excluded the participant with ASD who failed to follow the instruction to press the button but still watched the visual presentation (F(2, 58) = 4.6, ε = 0.88, *p* < 0.02).

The histogram plot depicting individual variations in gamma VSPF modulation range corrected for age visualizes clear-cut between-group difference on the individual basis (Fig. 4b). While VSPF modulation range in approximately half of the individuals from the TD sample (11 from 19 subjects) exceeded 30 Hz, it was below 30 Hz in all participants from the ASD sample. Vice versa, the VSPF modulation range less than 20 Hz was disproportionally increased in the ASD comparing to the TD group (10 from 16 ASD and 2 from 19 TD subjects). Thus, despite a partial overlap in the VSPF modulation range values between the two samples, the velocity-related growth of HGO frequency was obviously abnormally reduced in participants with ASD.

### Age dependence of gamma frequency parameters in the ASD and TD groups

Our previous study has shown reliable developmental changes in gamma VSPF and its modulation range in the TD boys [35]. Here, the significant GLM velocity by age interaction effect (F(2,64) = 3.66; ε = 0.88; *p* < 0.05) was due to the developmental slowing of VSPF under slow and medium velocity conditions and the absence of such slowing under the fast velocity conditions. We further computed Spearman correlations between gamma frequency parameters and subject’s age separately in the TD and ASD groups. For the both groups, the gamma VSPF measured under conditions of slow and medium velocities correlated negatively with age (slow velocity: both *Rho* < –0.6; both *p* < 0.01; medium velocity: both *Rho* < –0.5; both *p* < 0.03), whereas no significant correlation with age was found for fast velocity in either TD or ASD children. The gamma modulation range significantly increased with age in the TD group (*Rho* = 0.45; *p* < 0.05), but not in the ASD group (*Rho* = 0.16; ns). Homogeneity of slope analysis confirmed that the slopes of the regression lines (VSPF modulation range vs. age) significantly differed between the TD and ASD groups (age × group: F(1,31) = 7.4; *p* < 0.02.

### Gamma VSPF modulation range, IQ, and severity of autism

**Fig. 5:**
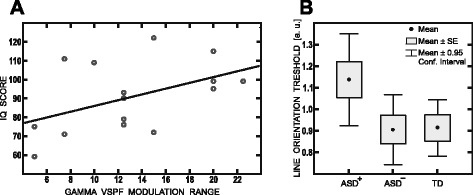
Correlation between gamma VSPF modulation range and psychological variables. **a** Scatter plots of correlation results for gamma VSPF and IQ in the ASD group. **b** Comparison of the oblique line discrimination thresholds in TD children and ASD children with (ASD+) and without (ASD−) marked reduction of gamma VSPF modulation range. Thresholds are given in the arbitrary units (a.u.) representing log-rescaled values of degrees of visual angle

The reduced range and the abnormal age dynamics of gamma frequency modulations in ASD raise a question of possible relation between gamma VSPF modulation range and severity of autism symptoms and/or the degree of developmental delay in ASD. The marginally significant correlation between VSPF modulation range and IQ (Spearman’s *Rho* = 0.49; *p* = 0.05) suggests that the reduced VSPF modulation range in children with ASD is related to developmental delay (Fig. 5a). No correlation between VSPF modulation range and AQ was found in the ASD group (*Rho* = –0.21; *p* = 0.41).

### Gamma VSPF modulation range and visual orientation discrimination threshold

At the next step, we investigated the relation between velocity-induced gamma frequency modulation and performance of the visual orientation discrimination task. All the measures (oblique line orientation threshold and VSPF at three stimulus velocities) were available for 13 and 16 subjects from the ASD and TD groups, respectively. We repeated the previous GLM analysis for this smaller sample. The group × velocity interaction remained significant (F(2,50) = 5.08; ε = 0.94; *p* < 0.02) ensuring that the selected sample demonstrated characteristic between-group difference in VSPF modulation range.

The oblique line orientation threshold did not differ between ASD and TD participants (F(1,27) = 1.27; *p* = 0.22). We further performed the regression analyses to test whether gamma VSPF or its modulation range predicted subjects’ orientation discrimination threshold. Age and IQ were also included as regressors into the analyses. In the TD group, oblique line orientation threshold was predicted neither by VSPFs nor by VSPF modulation range (for all partial correlation *p*’s > 0.5). In the ASD group, the regression model, which included VSPF modulation range, accounted for 59 % of the threshold variance (F(3,9) = 5,93; *p* < 0.017; age β = –0.38; *p* = 0.08; gamma modulation range β = –0.59; *p* <0.03; IQ β = –0.12; ns). Non-parametric correlation between oblique line orientation threshold and VSPF modulation range was also significant (*Rho* = –0.72; *p* < 0.006). The regression model, which included VSPF, was significant only in case of the fast velocity (F(3,9) = 7,76; *p* < 0.01; age β = –0.65; *p* < 0.01; VSPF β = –0.63; *p* < 0.01; IQ β = –0.12; ns). However, the non-parametric correlation did not reach significance level (*Rho* = –0.55; *p* = 0.06). Thus, in ASD participants, a higher oblique line orientation threshold was predicted by a narrower gamma modulation range, and this relation was not explained by age or IQ.

To further investigate the relation between presence of considerable deficit in VSPF modulation range and oblique line orientation threshold in children with ASD, we split them into two sub-groups. The first sub-group included six ASD subjects who scored equal to or less than the median age-corrected VSPF modulation range (18.05 Hz). The second sub-group included seven ASD subjects whose values exceeded the group median. The third group comprised all 16 TD participants. Taking into account small and unequal sample sizes, we applied non-parametric Kruskal–Wallis one-way analysis of variance to test for the main effect of group on oblique line orientation threshold. The main effect was significant (Kruskal–Wallis test: H = 7.68; *p* < 0.022), and we further applied the Mann–Whitney test to compare the “deficient VSPF modulation range group” with the rest of the ASD sample as well as with the TD children. The results of this comparison are plotted in Fig. 5b. ASD children with marked gamma frequency modulation abnormalities had higher oblique line discrimination threshold than either control children (Z = 2.43, *p <* 0.03) or ASD children without such abnormalities (Z = 2.42; *p* < 0.02). We should note that considering the small sample size, the result of the Kruskal–Wallis analysis should be interpreted with caution and have to be verified in an independent sample.

Altogether, the results strongly suggest that atypically narrow gamma VSPF modulation range and abnormally poor ability to discriminate orientation of oblique lines are interrelated and characterize a substantial proportion of children with ASD.

## Discussion

The role the induced gamma oscillations play in sensory processing, as well as their neurochemical basis and putative functional consequences of their atypical dynamics in neuropsychiatric disorders, are the hot topics in the literature [56]. In this regard, our finding of the reduced modulation of HGO frequency by velocity of visual motion in children and adolescents with ASD is of considerable interest.

Our results show that while the velocity-specific peak frequency of gamma response within 50–120 Hz range in TD children increases with increasing velocity of visual motion, the substantial proportion of children with ASD demonstrate sharply reduced gamma frequency gain. The atypical reduction was observed in the form of abnormally decreased high-frequency gamma power at fast stimulus velocity in grand average spectra of gamma response (Fig. 2, right panel). It was also evident as the reduced gain of gamma VSPF following increase in stimulus velocity from 1.2 up to 6 °/s at both individual (Fig. 3e, f) and group (Fig. 4) levels. The range of velocity-related gamma frequency modulation correlated inversely with the ability to discriminate oblique line orientation in children with ASD, while no such correlation has been observed in the typically developing control children.

### Velocity-related modulations of HGO frequency

The reliable increase in power of visual gamma oscillations elicited by moving annular grating in children and adolescents (Fig. 1) is generally consistent with the previous findings in adults [25–27, 55] and children [35]. On the other hand, this is the first study that manipulated stimulus velocity in order to capture velocity-related changes in gamma frequency and to scrutinize a putatively deficient functional modulation of gamma frequency in ASD.

The common pattern across all our subjects was the robust monotonic increase of gamma oscillation frequency that paralleled the increase in drifting grating velocity from 1.2 to 6.0 °/s. This finding fully agrees with the animal data obtained from local field potentials (LFP) recordings in the visual cortex [34]. This strong velocity dependence of gamma response frequency is in stark contrast to the data from the human studies that demonstrated the lack of substantial changes in gamma frequency in response to experimental manipulations with stimulus size ([57], but see also [27]), contrast [58], or spatial frequency [59, 60]. This tempted the authors to conclude that MEG does not possess sufficient sensitivity to capture stimulus-driven gamma frequency changes exceeding the limit of 70 Hz, possibly because of small amplitude of such oscillations [55]. Our results challenge this view by showing that the visual gamma response within 70–95 Hz frequency range is readily picked up by occipital MEG sensors (Figs. 2 and 3).

In the recently published study, we discussed in details the normal developmental course of visual gamma oscillations as well as the mechanisms underlying stimulus-related changes in gamma frequency [35]. In particular, we speculated that the high frequency of gamma response in children in our study might result from the high velocity of visual motion used in our experimental paradigm as well as from developmental changes in excitability of the FS PV^+^ interneurons.

### Typical and atypical features of visual gamma frequency modulation in ASD children

Prior EEG studies of gamma oscillations in ASD have characterized the general trend toward abnormally increased or decreased power of visually triggered induced and evoked gamma-band oscillations [19–21, 61, 62]. In addition, atypically diminished modulations of high gamma (60–120 Hz) power in ASD were found when the researchers manipulated spatial frequency of visual gratings [63], amount of homogeneously oriented elements in the stimuli [64], or orientation of a face [62].

Contrary to our expectation, in case of the slow velocity (1.2 °/s), neither frequency nor power of velocity-specific (i.e., within 52.5–77.5 Hz range for this velocity) gamma response was affected in participants with ASD. In sharp contrast to a roughly normal velocity-specific gamma response at low stimulus velocity, gamma frequency shift at faster velocities was remarkably reduced in a substantial proportion of participants with ASD (Fig. 4a). Thus, for at least some children with ASD, the visually perceived environmental motion at fast speed seems to present an important barrier for effective communication between neuronal populations through synchronous high-frequency gamma oscillations.

This finding suggests existence of a pathological mechanism limiting velocity-induced gamma frequency growth in autism. Generation and maintenance of gamma oscillations are known to critically depend on networks of FS PV-sensitive GABAergic interneurons [65]. Neurophysiological studies suggest that frequency and power of gamma oscillations are controlled by relatively independent network mechanisms [66]. In contrast to gamma power, gamma frequency, which underpins timing of spike trains during gamma oscillations, is less related to the excitatory state of principle cells [31, 67] and seems to be determined mainly by a balance between *N*-methyl-d-aspartate (NMDA) glutamate receptor-related excitatory and GABA-A receptor-related tonic inhibitory processes on membrane of inhibitory neurons [31]. Increase in gamma oscillation frequency is shown to reflect increased excitatory influence on inhibitory FS PV^+^ cells through NMDA receptors (NMDAR), causing faster kinetics of these cells and reducing time window of their synchronous discharge [31, 68]. Hence, faster velocities of drifting gratings in our study might lead to stronger excitation of critical FS PV^+^ inhibitory cells, thus accelerating frequency of gamma oscillations in the visual cortex. Given that NMDA-related excitability of inhibitory cells plays a crucial role in gamma-band frequency control, a plausible explanation of difficulties with velocity-related gamma frequency growth in children with ASD is the abnormal reduction of NMDAR-dependent excitatory modulation of FS PV^+^ inhibitory neurons.

Our hypothesis about NMDAR hypofunction on the FS PV^+^ interneurons as a putative cause of gamma frequency modulation difficulties in ASD is compatible with several lines of evidence.

Firstly, Billingslea et al. [6] have demonstrated that reduced FS PV-selective NMDAR signaling in the transgenic mice model closely mimics core autism features of reduced sociability and vocalizations as well as repetitive behavior [6]. In Shank2-mutant mice exhibiting ASD-like behaviors and carrying a mutation similar to the ASD-associated microdeletion in the human SHANK2 gene, NMDA agonists normalize NMDAR function and improve social interaction [69]. Neurophysiological study of Gandal et al. [70] revealed that constitutive NMDAR hypofunction of FS PV^+^ interneurons in transgenic mice induced multiple selective GABA-A receptors deficit on principle cells, increased intrinsic excitability of pyramidal cells, and selectively disrupted parvalbumin-expressing interneurons [70]. These changes resemble global alterations in the GABA-A receptor system and the reduction in parvalbumin containing interneurons in cingulum cortex that have been reported in postmortem studies of brain tissue in ASD [71].

Secondly, and most importantly for the proposed interpretation, recent work has shown that downregulation of NMDA transmission with NMDA receptor antagonists caused significant reduction in dominant frequency of high-frequency gamma oscillations induced by application of muscarinic acetylcholine receptor agonist to the slices of a rat’s primary visual cortex [68, 72]. The authors provide convincing arguments that NMDA receptor hypofunction decelerates peak frequency of induced high gamma oscillations by reducing activation of FS PV^+^ interneurons in superficial cortical layers II and III. Moreover, selective non-competitive NMDA receptor antagonist decelerated high-frequency gamma oscillations and left the dominant frequency of low frequency gamma unchanged.

The above experimental finding provides a parsimonious explanation for selective difficulties with visual gamma frequency modulation observed in a subset of children with ASD in our study. We speculate that dampened frequency increase of visual HGO response at higher velocities (3.6 and 6 °/s) in ASD is caused by hypofunction of NMDA receptors on FS PV^+^ cells in supragranular layers II and III in the visual cortex. Interestingly, the neurophysiological evidence on multiplicity of gamma-generating mechanisms in different layers of the visual cortex may partially explain presence of several spectral peaks with the similar peak frequencies at each stimulus velocity in the grand average spectra of power change in our study (Fig. 2). Experimental data from several studies suggest that higher-frequency gamma oscillations coexist with lower-frequency gamma oscillations, which are generated independently in the deeper infragranular layers V–VI of the visual cortex and do not react that much to the increasing strength of excitatory input [72]. Therefore, a spectrum of power change at each stimulus velocity in our study may, at least partially, reflect the relative contribution of different gamma generators. It is possible, that faster-drifting gratings produce greater involvement of HGO-driving circuitry in superficial layers II and III with concomitant increase in frequency and magnitude of respective gamma peak. Presence of abnormalities in layers II and III could explain the fact that abnormally decreased power change within the high-frequency part of the gamma spectrum (>80 Hz) was evident even at the lowest motion velocity in children with ASD (Fig. 2, 1.2 °/s: upper right panel).

The lack of the ASD/TD difference in power of velocity-specific gamma response at low stimulus velocity (Fig. 2) is, at the first glance, at odds with the proposed “NMDAR hypofunction” explanation. Indeed, genetic and optogenetic studies reported elevated background gamma power (“noise”) and reduced stimulus-induced gamma-band activity (“signal”) in mice with dysregulation of NMDA receptor signaling [70, 73]. This discrepancy between the proposed interpretation of our finding and animal data may be explained by a diversity of molecular pathways that can lead to disruptions in NMDAR signaling, with quite different effects on gamma oscillations and brain functions. It also seems unlikely that NMDAR activity in individuals with ASD is reduced to the same extent as in genetically modified mice. A more subtle disruption and/or selective, region or layer-specific abnormality of NMDA receptors may characterize brain pathology in children with ASD.

The above interpretation does not exclude the possibility that the local and large-scale network changes that can decelerate HGO may also contribute to the observed deficit in gamma frequency modulation in subjects with ASD. Specifically, fast speed of visual motion may increase gamma frequencies due to feedback to the primary visual cortex (V1) from higher-order areas, e.g., area MT of the dorsal visual steam, which exhibits higher sensitivity to faster moving stimuli than V1 [74]. In this case, difficulties with gamma frequency modulation in ASD could be caused by abnormal connectivity between V1 and higher-order visual areas [74]. However, Giselman and Tiel [75] pointed out that the feedback projections are not very likely sources of gamma frequency modulation in the primary visual cortex, since they target mainly pyramidal cells while the terminals on inhibitory interneurons are rare. Therefore, impairment of NMDA receptor-mediated transmission on GABAergic FS PV^+^ interneurons in V1 seems to be a more probable contributor to the observed abnormality in visual gamma frequency modulation in ASD.

### Psychophysical correlates of the altered gamma VSPF modulation range in ASD children

In the TD children, the oblique line orientation threshold correlated neither with individual gamma peak frequencies nor with velocity-related gamma frequency modulation. This finding is at odds with the results of Edden and colleagues, who demonstrated highly reliable inverse relationships between individual visual gamma peak frequency and oblique line orientation thresholds in typical adults [28]. This discrepancy might be explained by the young age of our subjects. Indeed, the developmental changes in myelination of cortical fibers, number of synapses, NMDA, α-amino-3-hydroxy-5-methyl-4-isoxazolepropionic acid (AMPA), and GABA neurotransmission all may influence gamma oscillations [56], affect individual gamma frequency, and explain age-dependant changes in its functional correlates. Indeed, in both the TD and ASD groups, we found reliable developmental decrease of VSPF at lower stimulus velocities. Obviously, the studies including multiple age groups may be necessary to narrow down the range of potential explanations for the discrepancy.

Unlike typically developing children, children with ASD demonstrated reliable inverse correlation between magnitude of gamma VSPF modulation and oblique line orientation threshold. We should stress that taken as a group, the participants with ASD did not differ significantly from the control participants in their performance on orientation discrimination task. However, the proportion of ASD children who were characterized by marked gamma frequency modulation difficulties did differ from controls and the rest of the ASD subjects by poor visual sensitivity to changes in oblique line orientation (Fig. 5b). Since both gamma frequency and the psychophysical index are thought to depend on functioning of inhibitory networks in the visual cortex, our findings show that altered visual functioning and neural oscillatory abnormalities in a subset of ASD children are related to impairment in this common circuit mechanism.

Edden and colleagues suggested that individual diversity in the frequency of visual gamma-band response and variation in orientation discrimination threshold may be both related to differences in the strength of GABAergic inhibition in V1 [28]. However, it is unlikely that the narrow gamma frequency modulation range in the affected ASD subjects could be explained by putative impairments in GABA receptors and/or GABA concentration in the visual cortex. Firstly, the recent studies in humans have shown that pharmacological manipulations with GABAergic transmission did not affect frequency of visual gamma oscillations measured with MEG [26, 76]. Secondly, in vitro studies imply that any GABA-A receptor abnormality on interneurons should be accompanied by an *increase* in peak frequency of visual gamma oscillation induced by excitatory input to the visual cortex [30, 31], i.e., the gamma frequency changes exactly opposite to those we observed in children with ASD in the present study. Given that (1) acceleration of visual gamma oscillations is critically linked to NMDA receptor-mediated transmission on GABAergic interneurons, (2) selective activation of GABAergic interneurons in the mouse V1 enhances orientation selectivity [77], and (3) cortical NMDA receptors are essential for the maturation of orientation selectivity of V1 neurons [78], we speculate that both narrowing of gamma frequency modulation range and relatively elevated line orientation thresholds might represent some of the observable consequences of abnormally reduced excitability of FS PV^+^ inhibitory neurons in the visual cortex. We also propose that constitutive NMDA receptor hypofunction is a likely cause of such deficit.

The visual gamma frequency abnormalities we described may be related to the degree of NMDAR dysfunction on FS PV^+^ interneurons in the visual cortex but not in other cortical areas more directly related to social and/or cognitive function. Indeed, we have not found a correlation between visual gamma frequency modulation and AQ—a measure of social reciprocity—in participants with ASD. However, we observed a direct relationship between gamma frequency modulation difficulties and a degree of mental developmental delay in the ASD group (Fig. 5a). Evidence from animal models implies that the selective disruption of NMDAR signaling in FS PV^+^ interneurons throughout the brain produces impairments in learning and memory [73]. Therefore, we assume that a severe shortage of gamma frequency modulation in visual circuitry may, at least indirectly, reflect more widespread NMDA-related deficit in those ASD children who display both autism and mental delay.

An interesting question is whether the difficulties with appropriate modulation of visual gamma frequency by speed of visual motion are associated with specific impairment in speeded visual processing in people with ASD. The accurate and fast visual analysis of stimulus motion is fundamentally important for production and control of motor activity. Our stimulus display consisted of relatively large (18 ° of visual angle) contracting annular gratings that were similar (although smaller) to those previously used to investigate how changes in optic flow affected postural reaction in children with ASD [79, 80]. Optic flow is the pattern of dynamic visual information that is projected onto the retina whenever individuals move through the environment. The contracting/expanding annular gratings providing illusion of self-motion are usually used to imitate such optic flow changes. Gepner and Mestre reported that children with ASD and mental retardation are compromised in their motor response to large field optic flow, which normally triggers postural adjustments [79, 80]. Notably, this deficit has been observed only under a condition of fast speed of visual motion, while slower changes in optic flow gave rise to typical postural response [80]. The authors speculate that the atypical motor response to the optic flow by observers with autism and mental retardation may reflect suboptimal coupling between visual and motor system [79, 81], possibly because their motor systems receive atypical input from the visual processing stream. The question that remained unanswered was why these impairments are so sensitive to the speeded changes in optic flow. Our findings provide an insight into possible neural underpinning of this atypical visual-motor coupling. As Maier and colleagues have suggested [82], high-frequency gamma activity in the superficial cortical layers is primarily related to cortico-cortical processing, because efferent projections from these layers mainly target extra-striate visual areas. To be processed and responded accurately and in time, fast moving target requires faster and more precise inter-neuronal communication between cortical areas involved. It is likely that faster communication takes place at higher gamma frequencies. Failure to appropriately increase gamma frequency in parallel with accelerating changes in visual environment should lead to poor adaptation to the “moving too fast” environment in people with ASD. Future studies might compare ASD children with and without abnormal postural reactivity to optic flow to assess whether those who express velocity-related gamma modulation difficulties also exhibit atypical visual-motor coupling.

## Conclusions

Whereas previous research have linked ASD to the reduced or enhanced power of gamma oscillations induced by visual or auditory sensory input, no studies have investigated the relationship between this behavioral phenotype and gamma frequency modulation under increasing functional load. Manipulating the speed of visual motion, we uncovered a severe deficiency in induced gamma frequency modulation in a proportion of children and adolescence with ASD. We also demonstrated a link between shortage of gamma frequency modulation and atypical visual functioning in ASD. Our findings suggest that deficiency in speeded visual processing at the cortical level in individuals with ASD may serve as an indicator of reduced functionality of inhibitory mechanisms involved in such basic visual function as discrimination of line orientation. We speculate that in view of the animal data, the reduced modulation of gamma frequency by stimulus velocity is likely associated with constitutive dysfunction of NMDA receptor-mediated transmission on GABAergic interneurons in ASD. Our approach complements the recent attempts of neurochemical, genetic and optogenetic research to identify divergent molecular pathways and cellular mechanisms underlying common behavioral phenotype of ASD. Frequency modulations of the MEG visual gamma oscillations may provide important insights into neural deficits that follow dysregulation of NMDA receptor signaling on FS PV^+^ inhibitory circuitry in ASD and possibly other neuropsychiatric disorders (e.g., schizophrenia, certain forms of mental retardation).

## Additional files

Additional file 1: Table S1. Sensitivity and specificity of the AS and SCQ-lifetime scores. Additional file 2: Table S2. Demographic and behavioral information on children included in statistical analysis of velocity-specific peak frequency (VSPF), mean ± sd (range).

## Abbreviations

AMPA: α-amino-3-hydroxy-5-methyl-4-isoxazolepropionic acid
AQ: autism spectrum quotient
ASD: autism spectrum disorders
E/I balance: balance between excitation and inhibition
ECG: electrocardiogram
EOG: electrooculogram
FS: fast spiking
FWHM: full width at half maximum
GABA: gamma-amino-butyric-acid
GLM: general linear modeling
HGO: high-frequency gamma oscillations
LFP: local field potential
MEG: magnetoencephalography
NMDA: *N*-methyl-d-aspartate
NMDAR: NMDA receptor
PV^+^: parvalbumin-expressing
RT: response time
TD: typically developing
tSSS: spatiotemporal signal space separation method
V1: primary visual cortex
VSPF: velocity-specific peak frequency

## References

[1] Persico AM, Bourgeron T (2006). Searching for ways out of the autism maze: genetic, epigenetic and environmental clues. Trends Neurosci.

[2] Geschwind DH (2011). Genetics of autism spectrum disorders. Trends Cogn Sci.

[3] Gandal MJ, Edgar JC, Ehrlichman RS, Mehta M, Roberts TP, Siegel SJ (2010). Validating gamma oscillations and delayed auditory responses as translational biomarkers of autism. Biol Psychiatry.

[4] Pizzarelli R, Cherubini E (2011). Alterations of GABAergic signaling in autism spectrum disorders. Neural Plast..

[5] Rubenstein JL, Merzenich MM (2003). Model of autism: increased ratio of excitation/inhibition in key neural systems. Genes Brain Behav.

[6] Billingslea EN, Tatard-Leitman VM, Anguiano J, Jutzeler CR, Suh J, Saunders JA (2014). Parvalbumin cell ablation of NMDA-R1 causes increased resting network excitability with associated social and self-care deficits. Neuropsychopharmacology.

[7] Oblak A, Gibbs TT, Blatt GJ (2009). Decreased GABAA receptors and benzodiazepine binding sites in the anterior cingulate cortex in autism. Autism Res.

[8] Siegel M, Donner TH, Engel AK (2012). Spectral fingerprints of large-scale neuronal interactions. Nat Rev Neurosci.

[9] Vinck M, Womelsdorf T, Fries P. Gamma-band synchronization and information transmission. In: Quiroga R-Q, Panzeri S, editors. Principles of Neural Coding. CRC Press; Boca Raton, FL, USA; 2013. p. 449-69.

[10] Bauer M, Oostenveld R, Peeters M, Fries P (2006). Tactile spatial attention enhances gamma-band activity in somatosensory cortex and reduces low-frequency activity in parieto-occipital areas. J Neurosci.

[11] Tallon-Baudry C, Bertrand O (1999). Oscillatory gamma activity in humans and its role in object representation. Trends Cogn Sci.

[12] Cheyne D, Bells S, Ferrari P, Gaetz W, Bostan AC (2008). Self-paced movements induce high-frequency gamma oscillations in primary motor cortex. Neuroimage.

[13] Muthukumaraswamy SD (2010). Functional properties of human primary motor cortex gamma oscillations. J Neurophysiol.

[14] Simmons DR, Robertson AE, McKay LS, Toal E, McAleer P, Pollick FE (2009). Vision in autism spectrum disorders. Vision Res.

[15] Liu BH, Li P, Sun YJ, Li YT, Zhang LI, Tao HW (2010). Intervening inhibition underlies simple-cell receptive field structure in visual cortex. Nat Neurosci.

[16] Zeng C, Li Y, Li C (2011). Center-surround interaction with adaptive inhibition: a computational model for contour detection. Neuroimage.

[17] Liu L, Pack C (2014). Bidirectional manipulation of GABAergic inhibition in MT: a comparison of neuronal and psychophysical performance. J Vis..

[18] Self MW, van Kerkoerle T, Super H, Roelfsema PR (2013). Distinct roles of the cortical layers of area V1 in figure-ground segregation. Curr Biol.

[19] Brown C, Gruber T, Boucher J, Rippon G, Brock J (2005). Gamma abnormalities during perception of illusory figures in autism. Cortex.

[20] Grice SJ, Spratling MW, Karmiloff-Smith A, Halit H, Csibra G, de Haan M (2001). Disordered visual processing and oscillatory brain activity in autism and Williams syndrome. Neuroreport.

[21] Stroganova TA, Orekhova EV, Prokofyev AO, Tsetlin MM, Gratchev VV, Morozov AA (2012). High-frequency oscillatory response to illusory contour in typically developing boys and boys with autism spectrum disorders. Cortex.

[22] Wright B, Alderson-Day B, Prendergast G, Bennett S, Jordan J, Whitton C (2012). Gamma activation in young people with autism spectrum disorders and typically-developing controls when viewing emotions on faces. PLoS One.

[23] Whitham EM, Pope KJ, Fitzgibbon SP, Lewis T, Clark CR, Loveless S (2007). Scalp electrical recording during paralysis: quantitative evidence that EEG frequencies above 20 Hz are contaminated by EMG. Clin Neurophysiol.

[24] Yuval-Greenberg S, Tomer O, Keren AS, Nelken I, Deouell LY (2008). Transient induced gamma-band response in EEG as a manifestation of miniature saccades. Neuron.

[25] Hoogenboom N, Schoffelen JM, Oostenveld R, Parkes LM, Fries P (2006). Localizing human visual gamma-band activity in frequency, time and space. Neuroimage.

[26] Muthukumaraswamy SD, Singh KD (2013). Visual gamma oscillations: the effects of stimulus type, visual field coverage and stimulus motion on MEG and EEG recordings. Neuroimage..

[27] van Pelt S, Boomsma DI, Fries P (2012). Magnetoencephalography in twins reveals a strong genetic determination of the peak frequency of visually induced gamma-band synchronization. J Neurosci.

[28] Edden RA, Muthukumaraswamy SD, Freeman TC, Singh KD (2009). Orientation discrimination performance is predicted by GABA concentration and gamma oscillation frequency in human primary visual cortex. J Neurosci.

[29] Cousijn H, Haegens S, Wallis G, Near J, Stokes MG, Harrison PJ (2014). Resting GABA and glutamate concentrations do not predict visual gamma frequency or amplitude. Proc Natl Acad Sci U S A.

[30] Ferando I, Mody I (2015). In vitro gamma oscillations following partial and complete ablation of delta subunit-containing GABAA receptors from parvalbumin interneurons. Neuropharmacology..

[31] Mann EO, Mody I (2010). Control of hippocampal gamma oscillation frequency by tonic inhibition and excitation of interneurons. Nat Neurosci.

[32] Kaiser J, Lutzenberger W (2005). Human gamma-band activity: a window to cognitive processing. Neuroreport.

[33] Eckhorn R, Frien A, Bauer R, Woelbern T, Kehr H (1993). High frequency (60-90 Hz) oscillations in primary visual cortex of awake monkey. Neuroreport.

[34] Gray CM, Viana Di Prisco G (1997). Stimulus-dependent neuronal oscillations and local synchronization in striate cortex of the alert cat. J Neurosci.

[35] Orekhova EV, Butorina A, Sysoeva OV, Prokofyev A, Nikolaeva A, Stroganova TA. Frequency of gamma oscillations in humans is modulated by velocity of visual motion. J Neurophysiol. 2015:jn 00232 2015. doi:10.1152/jn.00232.2015. 10.1152/jn.00232.2015 PMC4507959 25925324

[36] Muthukumaraswamy SD, Singh KD, Swettenham JB, Jones DK (2010). Visual gamma oscillations and evoked responses: variability, repeatability and structural MRI correlates. Neuroimage.

[37] Shaw ME, Hamalainen MS, Gutschalk A (2013). How anatomical asymmetry of human auditory cortex can lead to a rightward bias in auditory evoked fields. Neuroimage..

[38] Hata Y, Tsumoto T, Sato H, Hagihara K, Tamura H (1988). Inhibition contributes to orientation selectivity in visual cortex of cat. Nature.

[39] Kaufman AS, Kaufman NL (2004). Kaufman assessment battery for children second edition.

[40] Auyeung B, Baron-Cohen S, Wheelwright S, Allison C (2008). The autism spectrum quotient: children’s version (AQ-child). J Autism Dev Disord.

[41] Baron-Cohen S, Hoekstra RA, Knickmeyer R, Wheelwright S (2006). The autism-spectrum quotient (AQ)—adolescent version. J Autism Dev Disord.

[42] Rutter M, Bailey A, Lord C (2003). The social communication questionnaire (SCQ).

[43] Bloss CS, Courchesne E (2007). MRI neuroanatomy in young girls with autism: a preliminary study. J Am Acad Child Adolesc Psychiatry.

[44] Giarelli E, Wiggins LD, Rice CE, Levy SE, Kirby RS, Pinto-Martin J (2010). Sex differences in the evaluation and diagnosis of autism spectrum disorders among children. Disabil health J.

[45] Taulu S, Kajola M, Simola J (2004). Suppression of interference and artifacts by the signal space separation method. Brain Topogr.

[46] Tadel F, Baillet S, Mosher JC, Pantazis D, Leahy RM (2011). Brainstorm: a user-friendly application for MEG/EEG analysis. Comput Intell Neurosci..

[47] Tesche CD, Uusitalo MA, Ilmoniemi RJ, Huotilainen M, Kajola M, Salonen O (1995). Signal-space projections of MEG data characterize both distributed and well-localized neuronal sources. Electroencephalogr Clin Neurophysiol.

[48] Uusitalo MA, Ilmoniemi RJ (1997). Signal-space projection method for separating MEG or EEG into components. Med Biol Eng Comput.

[49] Litvak V, Mattout J, Kiebel S, Phillips C, Henson R, Kilner J (2011). EEG and MEG data analysis in SPM8. Comput Intell Neurosci..

[50] Thomson DJ (1982). Spectrum estimation and harmonic analysis. Proc IEEE.

[51] Holland PW, Welsch RE (1977). Robust regression using iteratively re-weighted least-squares. Commun Stat - Theor M.

[52] Litvak V, Eusebio A, Jha A, Oostenveld R, Barnes G, Foltynie T (2012). Movement-related changes in local and long-range synchronization in Parkinson's disease revealed by simultaneous magnetoencephalography and intracranial recordings. J Neurosci.

[53] Nichols TE, Holmes AP (2002). Nonparametric permutation tests for functional neuroimaging: a primer with examples. Hum Brain Mapp.

[54] Rinehart NJ, Bradshaw JL, Brereton AV, Tonge BJ (2001). Movement preparation in high-functioning autism and Asperger disorder: a serial choice reaction time task involving motor reprogramming. J Autism Dev Disord.

[55] Swettenham JB, Muthukumaraswamy SD, Singh KD (2009). Spectral properties of induced and evoked gamma oscillations in human early visual cortex to moving and stationary stimuli. J Neurophysiol.

[56] Uhlhaas PJ, Pipa G, Neuenschwander S, Wibral M, Singer W (2011). A new look at gamma? High- (>60 Hz) gamma-band activity in cortical networks: function, mechanisms and impairment. Prog Biophys Mol Biol.

[57] Perry G, Hamandi K, Brindley LM, Muthukumaraswamy SD, Singh KD (2012). The properties of induced gamma oscillations in human visual cortex show individual variability in their dependence on stimulus size. Neuroimage..

[58] Hall SD, Holliday IE, Hillebrand A, Singh KD, Furlong PL, Hadjipapas A (2005). The missing link: analogous human and primate cortical gamma oscillations. Neuroimage.

[59] Hadjipapas A, Adjamian P, Swettenham JB, Holliday IE, Barnes GR (2007). Stimuli of varying spatial scale induce gamma activity with distinct temporal characteristics in human visual cortex. Neuroimage.

[60] Muthukumaraswamy SD, Singh KD (2008). Spatiotemporal frequency tuning of BOLD and gamma band MEG responses compared in primary visual cortex. Neuroimage.

[61] Gross E, El-Baz AS, Sokhadze GE, Sears L, Casanova MF, Sokhadze EM (2012). Induced EEG gamma oscillation alignment improves differentiation between autism and ADHD group responses in a facial categorization task. J Neurother.

[62] Sun L, Grutzner C, Bolte S, Wibral M, Tozman T, Schlitt S (2012). Impaired gamma-band activity during perceptual organization in adults with autism spectrum disorders: evidence for dysfunctional network activity in frontal-posterior cortices. J Neurosci.

[63] Milne E, Scope A, Pascalis O, Buckley D, Makeig S (2009). Independent component analysis reveals atypical electroencephalographic activity during visual perception in individuals with autism. Biol Psychiatry.

[64] Snijders TM, Milivojevic B, Kemner C (2013). Atypical excitation-inhibition balance in autism captured by the gamma response to contextual modulation. Neuroimage Clin..

[65] Bartos M, Vida I, Jonas P (2007). Synaptic mechanisms of synchronized gamma oscillations in inhibitory interneuron networks. Nat Rev Neurosci.

[66] Jia X, Xing D, Kohn A (2013). No consistent relationship between gamma power and peak frequency in macaque primary visual cortex. J Neurosci.

[67] Towers SK, Gloveli T, Traub RD, Driver JE, Engel D, Fradley R (2004). Alpha 5 subunit-containing GABAA receptors affect the dynamic range of mouse hippocampal kainate-induced gamma frequency oscillations in vitro. J Physiol.

[68] Anver H, Ward PD, Magony A, Vreugdenhil M (2010). NMDA receptor hypofunction phase couples independent gamma-oscillations in the rat visual cortex. Neuropsychopharmacology.

[69] Won H, Lee HR, Gee HY, Mah W, Kim JI, Lee J (2012). Autistic-like social behaviour in Shank2-mutant mice improved by restoring NMDA receptor function. Nature.

[70] Gandal MJ, Sisti J, Klook K, Ortinski PI, Leitman V, Liang Y (2012). GABAB-mediated rescue of altered excitatory-inhibitory balance, gamma synchrony and behavioral deficits following constitutive NMDAR-hypofunction. Transl Psychiatry..

[71] Oblak AL, Gibbs TT, Blatt GJ (2010). Reduced GABAA receptors and benzodiazepine binding sites in the posterior cingulate cortex and fusiform gyrus in autism. Brain Res..

[72] Oke OO, Magony A, Anver H, Ward PD, Jiruska P, Jefferys JG (2010). High-frequency gamma oscillations coexist with low-frequency gamma oscillations in the rat visual cortex in vitro. Eur J Neurosci.

[73] Carlen M, Meletis K, Siegle JH, Cardin JA, Futai K, Vierling-Claassen D (2012). A critical role for NMDA receptors in parvalbumin interneurons for gamma rhythm induction and behavior. Mol Psychiatry.

[74] Priebe NJ, Lisberger SG, Movshon JA (2006). Tuning for spatiotemporal frequency and speed in directionally selective neurons of macaque striate cortex. J Neurosci.

[75] Gieselmann MA, Thiele A (2008). Comparison of spatial integration and surround suppression characteristics in spiking activity and the local field potential in macaque V1. Eur J Neurosci.

[76] Saxena N, Muthukumaraswamy SD, Diukova A, Singh K, Hall J, Wise R (2013). Enhanced stimulus-induced gamma activity in humans during propofol-induced sedation. PLoS One.

[77] Lee SH, Kwan AC, Zhang S, Phoumthipphavong V, Flannery JG, Masmanidis SC (2012). Activation of specific interneurons improves V1 feature selectivity and visual perception. Nature.

[78] Ramoa AS, Mower AF, Liao D, Jafri SI (2001). Suppression of cortical NMDA receptor function prevents development of orientation selectivity in the primary visual cortex. J Neurosci.

[79] Gepner B, Mestre D (2002). Rapid visual-motion integration deficit in autism. Trends Cogn Sci.

[80] Gepner B, Mestre DR (2002). Brief report: postural reactivity to fast visual motion differentiates autistic from children with Asperger syndrome. J Autism Dev Disord.

[81] Gepner B, Mestre D, Masson G, de Schonen S (1995). Postural effects of motion vision in young autistic children. Neuroreport.

[82] Maier A, Adams GK, Aura C, Leopold DA (2010). Distinct superficial and deep laminar domains of activity in the visual cortex during rest and stimulation. Front Syst Neurosci..

