# Supplementary material for: Altered modulation of gamma oscillation frequency by speed of visual motion in children with autism spectrum disorders
Source: J Neurodev Disord. 2015 Aug 10;7(1):21. doi: 10.1186/s11689-015-9121-x (PMC4530485; doi:10.1186/s11689-015-9121-x)
Supplement: Additional file 1: Table S1. — Sensitivity and specificity of the AS and SCQ-lifetime scores. [file 11689_2015_9121_MOESM1_ESM.docx]

Supplementary table 1. Sensitivity and specificity of the AS and SCQ-lifetime scores

|  | ASD mean (sd) | TD  mean (sd) | t | p | Sensitivity^2^ | Specificity^2^ |
| --- | --- | --- | --- | --- | --- | --- |
| AQ^1^ | 87.9±10.6  (N=19) | 56.4±15.1  (N=22) | t(39)=7.6 | <1e-5 | 95.5% | 91% |
| SCQ* | 25.1±4.7  (N=16) | 8.5±4.4  (N=21) | t(35)=11.0 | <1e-5 | 100% | 90% |

Notes.

1. Although the child [1] and adolescent [2] versions of AQ include the same questions, the details of scoring and presentation are somewhat different for children and adolescents. Since our study included participants from 7 to 15 years, to facilitate the ASD vs TD group comparison across ages we asked all the parents to fill in the child version of the questionnaire and used the child-type scoring for all the participants.

2. The Sensitivity and Specificity of the results are reported for the cut-off values used in the original publications (i.e. 76 for child AQ and 15 for SCQ-life). The previously reported values for the child AQ are 95% sensitivity and 95% specificity, when children with ASD were compared to the general population [1]. In case of the SCQ-life, the Sensitivity and Specificity reported for the 15 points cut-off were correspondingly 85 % and 75% when the ASD children were compared with children with other diagnoses [3].

**References**

1. Auyeung B, Baron-Cohen S, Wheelwright S, Allison C. The Autism Spectrum Quotient: Children's Version (AQ-Child). J Autism Dev Disord. 2008;38(7):1230-40.

2. Baron-Cohen S, Hoekstra RA, Knickmeyer R, Wheelwright S. The Autism-Spectrum Quotient (AQ)--adolescent version. J Autism Dev Disord. 2006;36(3):343-50.

3. Rutter M, Bailey A, Lord C. The Social Communication Questionnaire (SCQ). Los Angeles: Western Psychological Services; 2003.
