# Supplementary material for: Altered modulation of gamma oscillation frequency by speed of visual motion in children with autism spectrum disorders
Source: J Neurodev Disord. 2015 Aug 10;7(1):21. doi: 10.1186/s11689-015-9121-x (PMC4530485; doi:10.1186/s11689-015-9121-x)
Supplement: Additional file 2: Table S2. — Demographic and behavioral information on children included in statistical analysis of velocity-specific peak frequency (VSPF), mean ± sd (range). [file 11689_2015_9121_MOESM2_ESM.docx]

Supplementary Table 2. Demographic and behavioral information for children included in statistical analysis of Velocity-Specific Peak Frequency (VSPF): mean±sd (range).

|  | **ASD** | **TD** |
| --- | --- | --- |
| **AGE (years)** | 10.3±1.9(7.9-13.8)  N=16 | 11.3±1.3(8.7-14.1)  N=19 |
| **Sequential IQ**^#^ | 91.6±15.4(63-112)  N=15 | **105.8±10.1(88-121)***  N=18 |
| **Simultaneous IQ**^#^ | 91.5±18.3(71-144)  N=15 | **120.7**±**13.9**(91-150**) ***  N=18 |
| **Mental Processing Composite**^#^ | 90.1±18.5(59-127)  N=15 | **117.6**±**12.4**(92-141**) ***  N=18 |
| **Child AQ**^##^ | 88.3±11.8(73-119)  N=15 | **54.8**±**15.8**(32-85**)***  N=17 |
| **Oblique orientation discrimination threshold, in degrees of visual angle**^###^ | 1.0±0.22(0.7-1.37)  N=13 | 0.91±0.24(0.2-1.24)  N=16 |

^#^IQ was available in 15 of 16 ASD and in 18 of 19 TD participants.

^##^AQ was available in 15 of 16 ASD and in 17 of 19 TD participants.

^###^ Oblique orientation discrimination thresholds were available in 13 of 16 ASD and in 16 of 19 TD participants.

* Statistically significant difference between the groups (T-test, p<0.05)
